# ﻿A review of the intertidal *Medon* Stephens (Coleoptera, Staphylinidae, Paederinae) with description of a new species on the East Asian coasts

**DOI:** 10.3897/zookeys.1226.137489

**Published:** 2025-02-06

**Authors:** Kee-Jeong Ahn, Gae-Nam Jeon

**Affiliations:** 1 Department of Biology, Chungnam National University, Daejeon 34134, Republic of Korea Chungnam National University Daejeon Republic of Korea

**Keywords:** *COI*, identification key, rove beetle, seashore, Staphylinoidea, taxonomy

## Abstract

A taxonomic review of the seashore-inhabiting *Medon* Stephens along the East Asian coasts is presented based on morphological and molecular characters (*COI*). Specimens of *Medoncalculosus* Ahn & Jeon, **sp. nov.** and *M.tomokoae* Shibata & Fujimoto are extremely similar to each other, although there are differences in the form and structure of the aedeagus. Detailed comparison of morphological characters and molecular analyses based on genetic divergence and gene tree monophyly for species delimitation support the validity of the new species. Intraspecific genetic divergence of *COI* using uncorrected p-distance among *Medon* individuals ranged from 0% to 0.79%, while interspecific divergence among three Korean *Medon* species ranged from 7.74% to 11.69%. Morphological characters of *M.calculosus* Ahn & Jeon, **sp. nov.**, *M.prolixus* (Sharp), and *M.tomokoae* are compared. The new species is described with illustrations of diagnostic characters and a key to species of the East Asian coastal *Medon* is presented.

## ﻿Introduction

The genus *Medon* Stephens contains four coastal species along the eastern Pacific, Indian, and northern Atlantic Oceans including the Mediterranean Sea. These species inhabit decaying seaweed on sandy or pebble/gravel beaches (Frank and Ahn 2011; Kim et al. 2011; Shibata and Fujimoto 2017).

As part of an ongoing taxonomic revision of Korean coastal Staphylinidae, we collected several specimens belonging to *Medon* using the floatation method on pebble/gravel beaches. They are very similar to *M.tomokoae* Shibata & Fujimoto from Japan, although there are differences in the external form and internal structure of the male genitalia. This led us to investigate the species delimitation of these *Medon* species in more detail using molecular criteria based on genetic divergence and gene tree monophyly based on *COI* sequences.

In this paper, we compare morphological and molecular characters among individuals of the East Asian coastal *Medon* species and describe *M.calculosus* Ahn & Jeon, sp. nov. with illustrations of diagnostic characters.

## ﻿Material and methods

The depositories of the specimens examined are as follows: Chungnam National University Insect Collection (CNUIC), Daejeon, Korea, and National Museum of Nature and Science (NMNS), Tsukuba, Japan.

Habitus photographs were made using a Canon EOS 5Ds with an attached Canon MP-E65 mm f/2.8 1–5x macro lens. The produced multilayered images were merged using software (Helicon Focus 7). Microphotographs of mouthparts, male genitalia, and abdominal segments were prepared using a Leica MC170 HD microscope camera mounted on an Olympus BX51 compound microscope. All photographs were finally edited in Adobe Photoshop CS4 and CC.

A total of eight new sequences were generated from the Korean specimens (633 bp of the partial *COI* gene region). All the new sequences were deposited in GenBank (accession numbers: PP578207– PP578214 in Table 2). For comparison, 45 partial *COI* (624 bp) sequences were downloaded from GenBank for five *Medon* species (accession numbers: MW259761, MN249800, KR129040, HQ953660, HQ953398, HQ954439, HQ954438, KR487620, KR491265, KU919263, KU919097, KU916556, KU915842, KU915543, KU913155, KU913130, KU912779, KU912022, KU911503, KU910374, KU909268, KU909128, KU908943, KU908290, KU907897, KM451678, KM449956, KM449805, KM449734, KM449603, KM448496, KM447995, KM447725, KM447712, KM447270, KM446843, KM446508, KM445710, KM445254, KM444731, KM444711, KM441191, KM441020, KM440342, and KJ962140). DNA extraction, sequencing, and alignment were performed with reference to Song et al. (2019). Primers and amplification strategies are detailed in Yoo et al. (2021).

Intra- and interspecific distances were calculated using the uncorrected pairwise distance method in MEGA 7.0 (Kumar et al. 2016). Parsimony (PA) and maximum likelihood (ML) analyses were conducted using PAUP* 4.0 (Swofford 2002) and PhyML 3.3 (Guindon et al. 2010) with default options implemented in Geneious (ver. 2025.0.2). Clade support values were evaluated using 100 bootstrap replicates.

## ﻿Taxonomy

### ﻿Key to species of the East Asian coastal *Medon*

**Table:** 

1	Body length more than 3.8 mm; body black with brown elytra; black tentorial spots absent on the vertex; antennomeres 8–10 subglobular, about 1.1 times as long as wide; hind wings fully developed and long, reaching to the end of abdomen; apical margin of male sternite VII protruded (Fig. 5A); male sternite VIII deeply emarginate (Fig. 5B)	***M.prolixus***
–	Body length less than 3.7 mm; body brown with black abdomen (Fig. 1A−C); black tentorial spots present on the vertex (Fig. 1A−C); antennomeres 8–10 subglobular, more than 1.2 times as long as wide; hind wings short, not reaching to the end of abdomen; apical margin of male sternite VII subtruncate (Fig. 3A); male sternite VIII slightly emarginate or subtruncate (Fig. 3B, 4B)	**2**
2	Apical margin of male sternite VIII subtruncate (Fig. 4B); aedeagus as in Fig. 4E, F (narrower in dorsal view, apical process shorter in lateral view, paramere smaller)	***M.tomokoae***
–	Apical margin of male sternite VIII slightly emarginate and with small sinuation at middle (Fig. 3B); aedeagus as in Fig. 3E, F (broader in dorsal view, apical process longer in lateral view, paramere larger)	***M.calculosus* sp. nov.**

### ﻿Genus *Medon* Stephens, 1833

#### ﻿ Medon calculosus sp. nov.

Taxon classificationAnimaliaColeopteraStaphylinidae

41D1757A-D8D9-54BB-9689-16BB9BC005BA

https://zoobank.org/0E15F233-F763-43A4-B30B-4D83C5D46B0A

Figs 1A, B , 2 , 3

##### Type specimens.

***Holotype*** • labeled as follows: “Korea: Gyeongbuk Prov., Pohang-si, Janggi-myeon, Gaewon-ri 445-12 35.855905°N, 129.524668°E, 31 X 2023, KJ Ahn, pebble beach in low-tide zone; Holotype, *Medoncalculosus* Ahn and Jeon, Desig. K.-J. Ahn 2024, deposited in CNUIC, Daejeon, Korea”. ***Paratypes*** • 3 exx., same data as holotype • 2 exx., Korea: Gyeongbuk Prov., Pohang-si, Janggi-myeon, Gaewon-ri 445-12 35.855905°N, 129.524668°E, 2 XI 2023, KJ Ahn, pebble beach in mid-tide zone • 2 exx., same locality as above, 24 III 2023, KJ Ahn, pebble beach in low-tide zone • 2 exx., Gyeongju-si, Yangnam-myeon, Suryeom-ri, Jigyeong-beach, 35.652814°N, 129.450041°E, 2 XI 2023, KJ Ahn, pebble beach in low-tide zone • 2 exx., Naa-ri, Naa-beach, 35°41′57.31″N, 129°28′26.73″E, 7m, 13 VII 2018, IS Yoo, JS Lee, JG Jung, in gravels covered with seaweed in high-supratidal zone, flotation • 2 exx., Yeongduk-gun, Byonggok-myeon, Byonggok-ri, 36.602581°N, 129.415695°E, 24 IX 2022, KJ Ahn, flotation on pebble beach.

##### Description.

**Figure 1.**
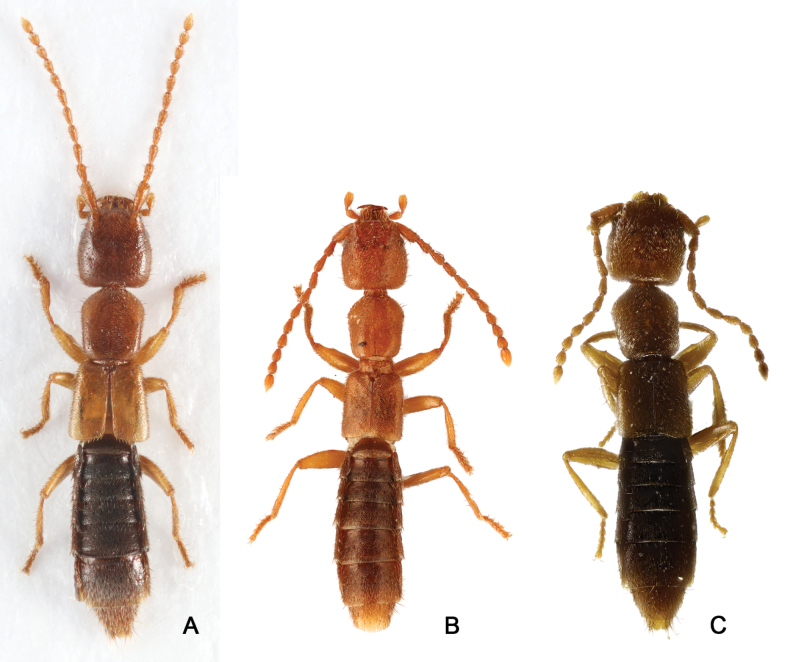
Male habitus **A***Medoncalculosus* sp. nov. 3.1 mm **B** paler form of *M.calculosus* sp. nov. **C***M.tomokoae*, 3.2 mm.

**Male.** Body length 3.0–3.3 mm. Body slender, more or less parallel-sided, flattened, and densely pubescent. Head, pronotum, elytra, antennae and legs brown to reddish-brown; abdomen black (Fig. 1). ***Head.*** Subquadrate, about 1.03 times as long as wide, widest basal 1/5 and about 1.15 times wider than pronotum, dorsal surface covered with dense pubescence; eye small, about 0.26 times as long as temple; antenna very long, extending to middle of elytron; inserted under side of front head, insertion invisible from dorsal view; all antennomeres elongate, scape longest and widest, pedicel to antennomere 7 distinctly longer than wide, antennomeres 8–10 subglobular, antennomere 11 water drop-shaped, relative length of 11 antennomeres 21:14:14:13:14:13:13:11:11:11:14; gular sutures narrowly separated and converged posteriorly. Neck moderately wide, about 1/3 as wide as head.

**Figure 2.**
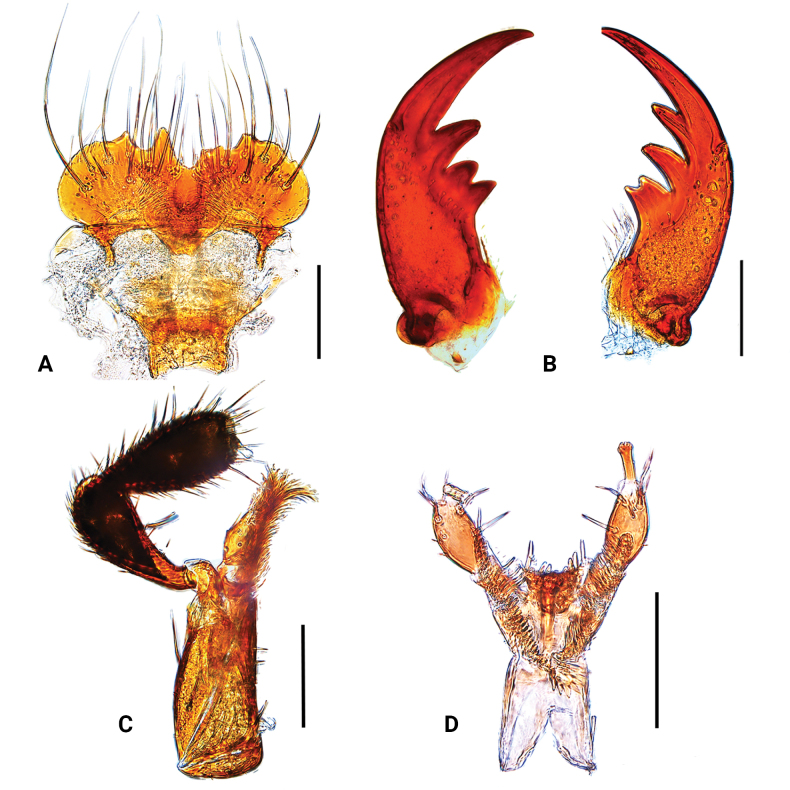
*Medoncalculosus* sp. nov., male **A** labrum, dorsal aspect **B** mandible, dorsal aspect (left and right) **C** maxilla, dorsal aspect **D** labium, ventral aspect. Scale bars: 0.1 mm.

**Figure 3.**
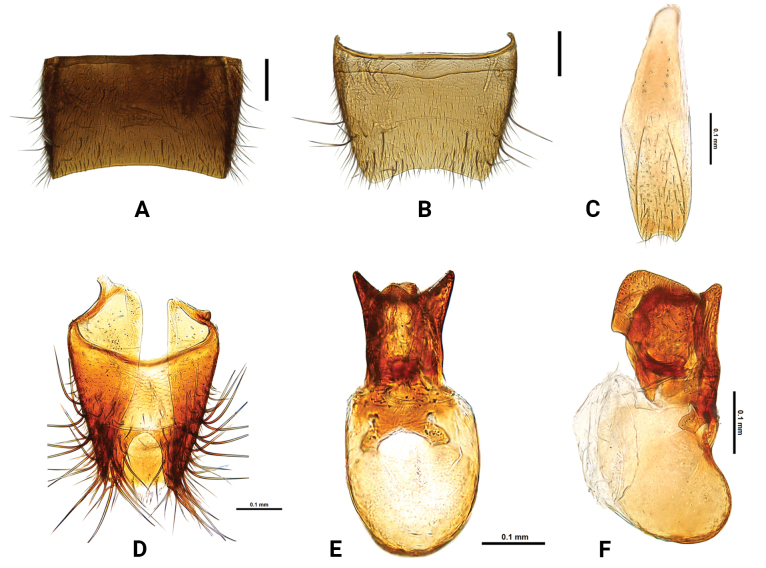
*Medoncalculosus* sp. nov. **A** male sternite VII, ventral aspect **B** male sternite VIII, ventral aspect **C** male sternite IX, ventral aspect **D** male tergites IX and X, dorsal aspect **E** aedeagus, dorsal aspect **F** aedeagus, lateral aspect. Scale bars: 0.1 mm.

***Mouthparts.*** Labrum (Fig. 2A) broad, anterior margin with two pairs of tooth-like processes, medial one longer and slender; mandibles (Fig. 2B) asymmetrical, with 3–4 internal teeth; maxilla (Fig. 2C) with galea and lacinia fringed with long setae; maxillary palpomere 1 small, about 3.18 times as long as 2; palpomere 2 expanded apically, little curved inwardly, about 0.97 times as long as 3; palpomere 3 droplet-shaped, widest apical third; palpomere 4 minute, about 0.88 times as long as 1; labium (Fig. 2D) with palpomere 1 small, about 0.46 times as long as 2; palpomere 2 widest near middle, about 1.87 times as long as 3; palpomere 3 small and thin, about 1.13 times as long as 1. ***Thorax.*** Pronotum more or less rectangular, about 1.17 times as long as wide; narrower than head, almost as long as head, widest apical 1/5 and narrowed posteriorly, yellow short setae densely present, impunctate central region present; prosternum well developed, median area upheaved with transverse carina, prosternal process acute and very long, hypomeral projection well developed and triangular. Elytra about 1.09 times as wide as pronotum, almost as long as pronotum, elytron 2.37 times as long as wide, distinct and shallow punctures present, covered with yellow setae, lateral margin straight and parallel. Metendosternite Y-shaped. Hind wings short but longer than elytron. Tarsal formula 5-5-5, front, middle and hind tarsomeres 1–5 each strongly widened. ***Abdomen.*** Almost parallel-sided and broadened posteriorly after segment VI. Tergites III–VI with shallow and transverse basal depression. Sternite III with basal transverse carina, medially pointed; apical margin of tergite VII subtruncate (Fig. 3A) and sternite VIII slightly emarginate (Fig. 3B); apical margin of sternite IX (Fig. 3C) slightly emarginate; tergites IX and X (Fig. 3D) covered with scattered setae, apical margin with numerous long and brown setae. ***Aedeagus.*** Median lobe symmetrical, comprising about half sclerotized part and membraneous part; more or less triangular lobe protruded in dorsal view (Fig. 3E); apical process narrow and well sclerotized in lateral view (Fig. 3F).

**Female.** Similar to male, but apical margin of sternite VIII rounded.

##### Distribution.

Korea (South).

##### Etymology.

The name is derived from the Latin *calculus* meaning ‘pebble’, referring to the species’ marine coastal habitat.

##### Remarks.

**Figure 4.**
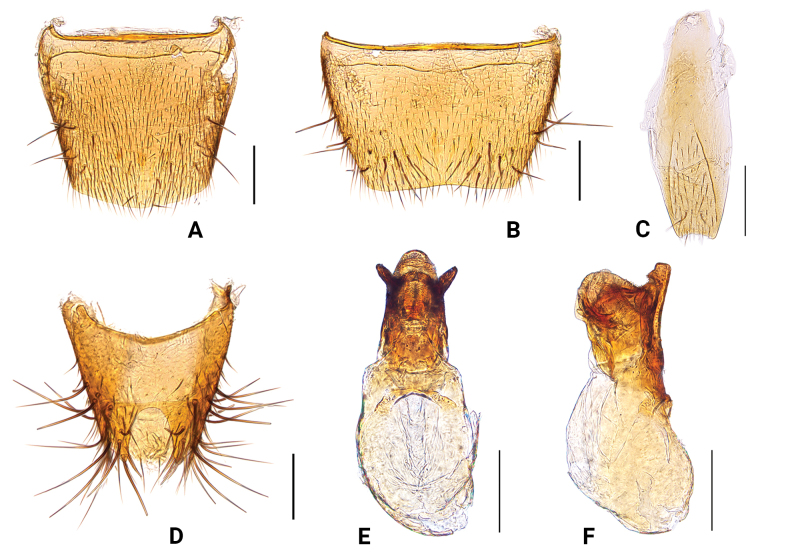
*Medontomokoae***A** male tergite VIII, dorsal aspect **B** male sternite VIII, ventral aspect **C** male sternite IX, ventral aspect **D** male tergites IX and X, dorsal aspect **E** aedeagus, dorsal aspect **F** aedeagus, lateral aspect. Scale bars: 0.1 mm.

This species is extremely similar to *M.tomokoae* in external morphological characters (Figs 3A–D, 4A–D) but can be distinguished by the different external form and internal structure of the aedeagus (Figs 3E, F, 4E, F) and by the characters listed in Table 1.

**Table 1.** Comparison of morphological characters between *Medoncalculosus* sp. nov., *M.tomokoae*, and *M.prolixus*.

	*M.calculosus* sp. nov.	*M.tomokoae*	*M.prolixus*
Body length	3.0–3.3 mm	2.8–3.5 mm	4.0–4.3 mm
Body color	brown to reddish-brown	brown to reddish-brown	black with brown elytra
Antenna length	very long, extending to the middle of elytron	very long, reaching to the middle of elytron	long, reaching to just behind the pronotum
Antennomeres	all elongate	all elongate	not all elongate, 8–10 subquadrate
Eye size	very small, 0.26 times as long as temple	very small, 0.28 times as long as temple	small, 0.4 times as long as temple
Black tentorial spots on vertex	present	present	absent
Pronotum	slightly narrowed posteriorly	slightly narrowed posteriorly	parallel-sided
Hind wings	short, less than 2.0 times as long as elytron	short, less than 2.0 times as long as elytron	long, more than 2.0 times as long as elytron
Male sternite VII	subtruncate (Fig. 3A)	subtruncate	protruded posteriorly (Fig. 5A)
Male sternite VIII	slightly emarginate (Fig. 3B)	subtruncate posteriorly (Fig. 4B)	deeply emarginate (Fig. 5B)
Median lobe in dorsal aspect	broader (Fig. 3E)	narrower (Fig. 4E)	narrower (Fig. 5C)
Median lobe in lateral aspect	apical process shorter (Fig. 3F)	apical process longer (Fig. 4F)	apical process shorter (Fig. 5D)
Microhabitats	pebble/gravel beach	pebble/gravel beach	sandy beach

**Table 2.** List of study species with their locality data and GenBank accession numbers.

Species	Collection locality	*COI*
*Medoncalculosus* sp. nov.	KOREA: Gyeongbuk, Gyeongju-si	PP578214
*Medonprolixus* 1	KOREA: Gyeongbuk, Uljin-gun	PP578207
*Medonprolixus* 2	KOREA: Gyeongbuk, Uljin-gun	PP578210
*Medonprolixus* 3	KOREA: Gyeongnam, Geoje-si	PP578211
*Medonprolixus* 4	KOREA: Gyeongnam, Tongyeong-si	PP578212
*Medonprolixus* 5	KOREA: Jeju Prov., Jeju-si	PP578213
*Medon* sp. 1A	KOREA: Gangwon, Sokcho-si	PP578208
*Medon* sp. 1B	KOREA: Jeonnam, Goheung-gun	PP578209

## ﻿Discussion

Diagnostic characters among members of the coastal *Medon* species (*M.calculosus*, *M.prolixus*, and *M.tomokoae*) include the shape of the antennomeres and male sternites VII–VIII, the size of the hind wings, body length, and the shape and structure of the male genitalia (Table 1). The male genitalia characters are especially important for species delimitation, as in other staphylinid beetles. Our morphological study showed that *M.calculosus* is clearly different from other previously described species.

Furthermore, the pairwise distance data support the validity of the new species: interspecific genetic divergence of the *COI* (633 bp) using uncorrected p-distance among eight *Medon* individuals, including one new species, ranged from 7.741% (between *M.prolixus* and *Medon* sp.) to 11.690% (*Medon* sp. and *M.calculosus*), while intraspecific divergence ranged from 0% to 0.790% (among *M.prolixus*) (Table 3). Our phylogenetic analyses also support the validity of the new species (Fig. 6). The PA tree showed polytomy in species relationships, but all seven species were supported as a lineage with 100% bootstrap value (not shown). One individual of *M.brunneus* (*M.brunneus* 1 – KR487620) and an unidentified specimen (*Medon* sp. – KR491265) were grouped with *M.fusculus* (Fig. 6). They need further investigation to confirm their identifications. Fresh specimens of *M.tomokoae* were not available for DNA analysis.

**Figure 5.**
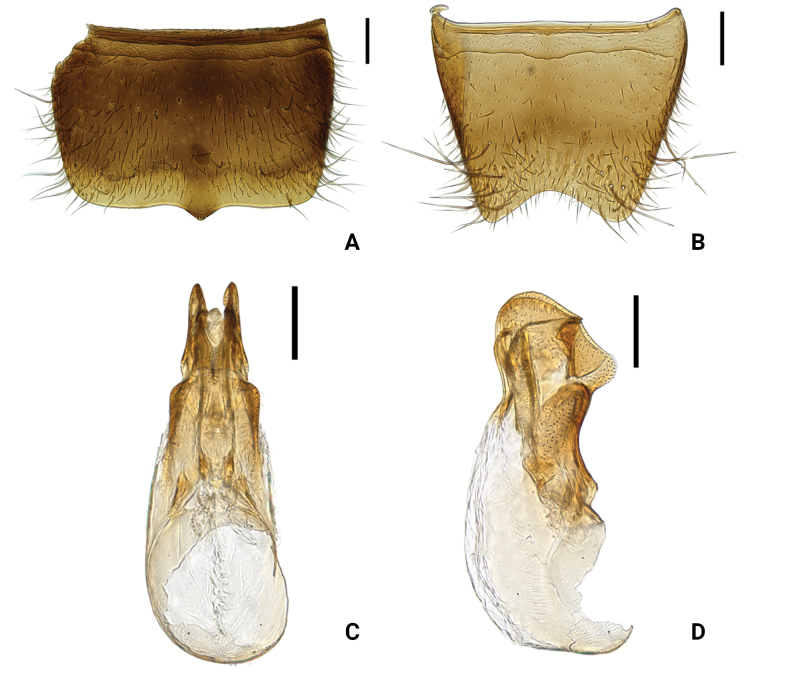
*Medonprolixus***A** male sternite VII, ventral aspect **B** male sternite VIII, ventral aspect **C** aedeagus, dorsal aspect **D** aedeagus, lateral aspect. Scale bars: 0.1 mm.

**Figure 6.**
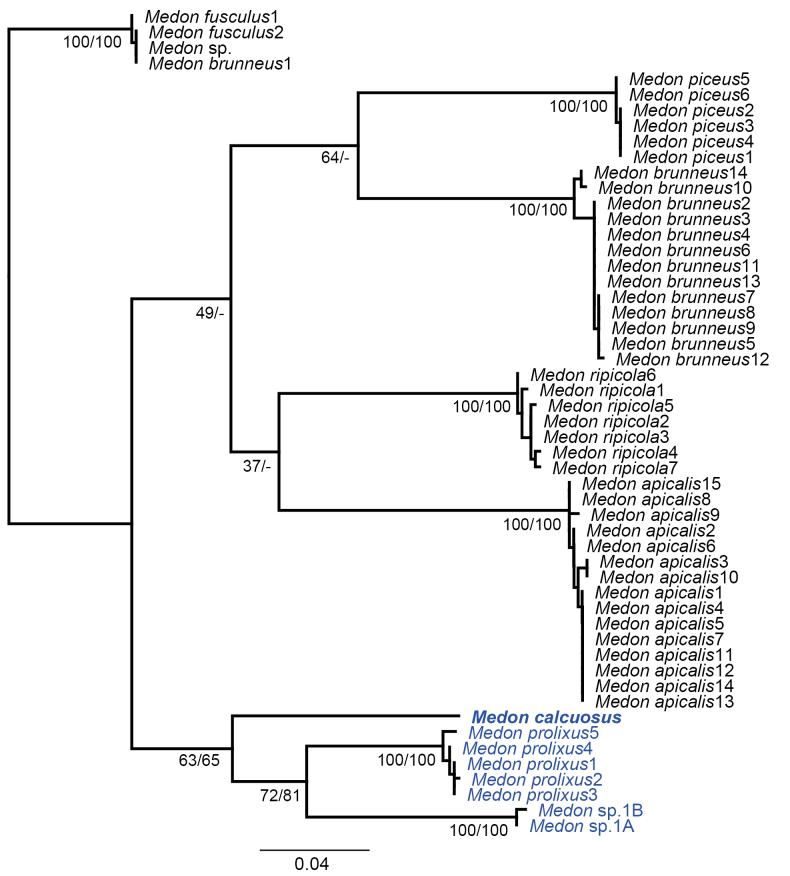
Maximum likelihood tree of *Medon* based on partial *COI* gene sequence with bootstrap values (left) and parsimony analysis bootstrap values (right).

**Table 3.** Inter- and intraspecific genetic differences in *Medon* species for *COI* (633 bp) calculated using p-distance.

	*M.calculosus* sp. nov.	*M.prolixus*	*Medon* sp. 1
*M.calculosus* sp. nov.	0	–	–
*M.prolixus*	9.795–10.111	0–0.948	–
*Medon* sp. 1	11.374–11.690	7.741–8.215	0.316

We found two specimens, one each from Sokcho (central Korea) and Goheung (southern Korea), that showed a genetic difference from all other included species (7.741%). They were also separated from other species in the phylogenetic analyses (Fig. 6). However, they are all females and will remain undescribed until we find a male to confirm whether it could be *M.tomokoae* or a new species.

Shibata and Fujimoto (2017) were the first to note that *M.tomokoae* was collected under stones on pebble/gravel beaches. *Medoncalculosus* is the second species collected from the same microhabitats in association with *Halorhadinus* Sawada, *Myrmecopora* Saulcy, *Physoplectus* Reitter, and *Giulianium* Moore, among others, in the low to mid-tide zones. In contrast, *M.prolixus* is readily found under seaweed/debris on sandy beaches in high-tide to splash zones.

## Supplementary Material

XML Treatment for Medon calculosus

## ﻿Additional information

### Author ORCIDs

Kee-Jeong Ahn https://orcid.org/0000-0003-4880-0931

### Data availability

All of the data that support the findings of this study are available in the main text.
